# Spatiotemporal variations in soil cultivable mycobiota at the Arava desert (Israel) along latitudinal and elevational gradients

**DOI:** 10.3934/microbiol.2018.3.502

**Published:** 2018-06-28

**Authors:** Isabella Grishkan

**Affiliations:** Institute of Evolution, University of Haifa, 199 Aba Khoushy Ave., Mount Carmel, Haifa 3498838, Israel

**Keywords:** desert soils, diversity, melanin-containing fungi, microfungal communities, spatiotemporal dynamics, thermotoletant mycobiota

## Abstract

Regional, local, and seasonal distribution of soil culturable microfungi in the Arava Valley, Israel, was examined along altitudinal and latitudinal gradients. A total of 198 species from 86 genera were isolated using the soil dilution plate method. Melanin-containing species with large multi-cellular spores dominated the majority of microfungal communities, while species with picnidial fruit bodies mostly prevailed in the northern part of the Arava Valley located at 190 m below sea level. Aspergilli (mainly *Aspergillus fumigatus*) and teleomorphic ascomycetes comprised the basic part of thermotolerant mycobiota obtained at 37 °C. The soil at the northern part of the desert held the highest number of microfungal isolates and, at the same time, was characterized by significantly lower species richness. The open sun-exposed localities harbored a significantly higher number of species than the localities under shrub canopies. Isolate density displayed the opposite trend and was significantly lower in the open than in shrub localities. The mycobiota characteristics such as species composition, contribution of major groupings to mycobiota structure, diversity level, and isolate density showed significant correlations with measured edaphic parameters—organic matter content, water content, pH, and especially, with electrical conductivity. Among the environmental aspects, locality position along altitudinal and latitudinal gradients accompanied by locality type (open sun-exposed or under shrubs), strongly influenced the community's characteristics, thus demonstrating the effect of the unique altitudinal position of the northern part of the Arava Valley as well as the ability of microfungal communities to be sensitive to the microscale environmental variability.

## 1. Introduction

It is now well established that the development and functioning of soil microbial communities are strongly affected by environmental factors at different geographic scales ([1] and references therein). For that reason, diversity and distribution of soil fungi in stressful habitats is an important topic to study because it can shed light on the mechanisms of survival and adaptation of microorganisms in extreme environmental conditions. Deserts represent such stressful habitats where severe climate and limited resources greatly influence biota formation [2].

Deserts cover more than 60% of Israeli territory [3]. Such massive distribution of the desert area in the country offers an excellent opportunity to study soil fungi and their adaptive strategies on a broad environmental scale: from the semi-arid to extremely arid regions, with annual rainfall ranging from 300 mm to 25 mm, respectively. The gradient also covers a range of elevation and vegetation diversity, and such combinations of different climatic, microclimatic, and edaphic factors can principally govern the structure of soil fungal communities [4].

Numerous mycological studies have been conducted in desert soils all over the world [4]–[14]. It has been shown that both taxonomic and functional diversity of soil fungi in the arid zone is highly dependent on water availability, temperature regime, and organic matter content ([15] and references therein). Because of high spatiotemporal heterogeneity in resource availability, fungal species richness in the desert soils may be greater than expected based solely on consideration of abiotic conditions [16],[17]. Our previous mycological studies of soils in different locations at the central and southern parts of the Negev desert, Israel [18]–[22], confirmed the above consideration. They showed that in spite of hostile conditions (high radiation, very high summer temperatures, water deficiency, and oligotrophic conditions), soils of the Negev desert maintained rich cultivable mycobiota—altogether, more than 400 species belonging to 132 genera. The Negev soil's microfungal communities displayed remarkable adaptive strategies to harsh climatic and edaphic conditions associated mainly with the composition of dominant groups of species.

The present mycological study was conducted in the soil of another type of Israeli desert—the Arava Valley. The Arava, located in the southeastern part of Israel, belongs to the longitudinal Syrian-African Rift Valley. This desert, which is more than 160 km in length, extends from the Dead Sea in the north (31°) to the Gulf of Eilat (Gulf of Aqaba) in the south (29.5°). The elevation of the valley varies from about 400 m below sea level at the Dead Sea area to 210 m a.s.l. in the region of Arvat Yafruq, in the center of the valley, and then decreases southward to sea level at Eilat (the Red Sea) [23]. The main goal of the study addresses the adaptive pattern of cultivable mycobiota in the soils of the Arava Valley. Although representing only a part of the whole mycobiota, a cultivable fraction constitutes significant and essential taxonomic and functional diversity in the soil [24]. I hypothesized that the unique geographical position (part of the Arava Valley is located below sea level) would affect the composition and structure of soil microfungal communities (reflected in the abundance of groupings with different life-history strategies), their diversity level, and the amount of microfungi at a macroscale—along elevational and latitudinal gradients. It was also expected, based on our previous findings that differences in microclimatic and edaphic conditions at different locality types—under canopy and open, would cause remarkable microscale variations in microfungal communities. To test the hypothesis, the following characteristics of the communities were examined for spatiotemporal dynamics: species composition, contribution of major groupings to mycobiota structure, diversity level, dominant groups of species, and isolate density. The effect of soil abiotic factors on the above characteristics was also estimated.

## 2. Materials and methods

### 2.1. Site description

The study was conducted along the elevational and latitudinal gradient of the Arava Valley. The climate of the Arava region is defined as hyper-arid [23],[25], with an annual precipitation of 15–50 mm and a decreasing trend in the amounts of annual rainfall [26]. Mean annual maximal temperature during the hottest month of July is 39.5 °C, mean annual minimal temperatures during the coldest month of January are 7.8 °C and 13.6 °C at Sapir (near the center of the valley) and Sedom (near the Dead Sea), respectively [23]. Annual potential evaporation is ∼3200 mm. The major part of the Arava Valley floor consists of stony coarse alluvial soil, mainly on gypseous chalk formations [27]. The very sparse vegetation of the Arava Valley is represented by dwarf shrubs such as *Nitraria retusa*, *Traganum nudatum*, *Haloxylon salicornicum*, *Anabasis articulata*, and *Zygophyllum dumosum* accompanied by rare *Acacia* trees [28].

### 2.2. Sampling

**Figure 1.**
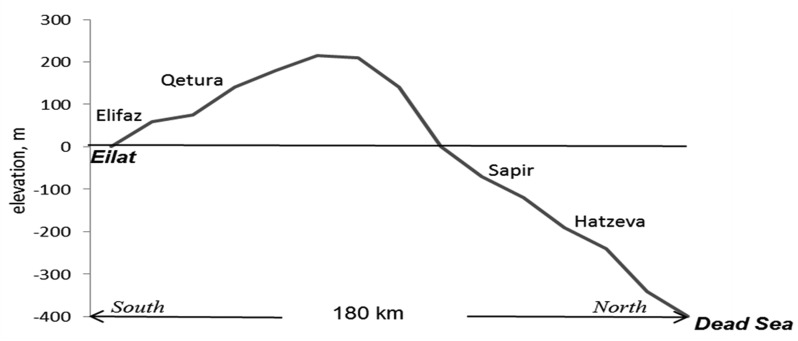
Location of sampling sites along the elevational and longitudinal gradients at the Arava Valley, Israel.

The sampling was conducted along the southward 120 km long transect of the Arava Valley. Soil samples were taken near the following settlements: Hatzeva (30°46′N 35°17′E; −190 m above sea level), Sapir (30°17′N, 35°11′E; −70 m a.s.l.), and Elifaz (29°48′N, 35°01′E; 60 m a.s.l.) (Figure 1). The samples for spatiotemporal analysis were collected in July 2014 and February 2015 from the upper soil layer (0–2 cm) at sunny-exposed, vegetation-free open localities and at localities under nearby shrubs (*Nitraria retusa*), from the area of approximately 250–500 m^2^—six samples from each locality at each sampling area and in each season, 72 samples altogether. At each micro-locality, 8 sub-samples from a plot of 20–20 cm were combined into a single pooled sample, 30–40 g. All samples, placed in sterile paper bags, were stored under dry conditions until processing (2–7 days).

Prior to the above samplings in July 2014 and February 2015, soil samples were collected in March 2011 and August 2011 at the same areas as well as near Ketura (29°58′N 35°4′E; 140 m a.s.l.) (Figure 1), 74 samples altogether. The samplings of July 2014 and February 2015 provided proper comparisons between the areas located at the northern, central, and southern parts of the desert with simultaneously different elevational positions—from 190 m below to 60 m above sea level, as well as between the locality types (open and under shrubs) and the contrasting seasons (summer and winter). Data from the samplings of March and August 2011 were additionally used for the general characterization of soil mycobiota of the Arava Valley. In order to reveal the effect of the unique, pronounced below-sea-level position of the northern sampling area (near Hatzeva), its microfungal communities were compared with communities isolated from the soil near Makhtesh Ramon (the central Negev desert)—located westward at a nearby latitudinal position (30°36′N) but at a height of 200–220 m a.s.l. [21].

### 2.3. Determination of soil temperature, moisture, pH, organic matter content, and electrical conductivity

Soil temperature was recorded at the depth of 0.5–1 cm with an E.T.I. 2001 thermometer (E.T.I. LTD, Sussex) at the time of sample collection, in order to characterize the differences between seasons (winter–summer) and between localities (open–under shrubs). Water content in soil samples (20–30 g) was determined gravimetrically as the loss in weight after heating at 105 °C for 24 h. Measurement of electrical conductivity was made in a water paste using Cyberscan con11 (EuTech Instruments, Landsmeer, The Netherlands). To create the paste, distilled water was added to 20 g of soil and left for 2 h to facilitate equilibrium between dissolved salts and water. The following analysis were performed in the Soil Survey Laboratory, Newe Ya'ar, Israel: pH measured in water paste (1 soil:2.5 water) by a digital pH meter; and organic matter content assessed by the Walkley-Black wet oxidation method with dichromate [29].

### 2.4. Characterization of fungal communities

For isolation of microfungi, the soil dilution plate method [30] was employed. Despite certain limitations and biases [31], this method “is simple and rapid, gives reasonable results, and yields excellent comparative data” and remains a useful approach for the initial characterization of the ecology of fungal communities [32]. It is especially applicable to desert soils where microfungi may exist for a long period in a dormant (spore) state.

Ten grams of soil samples were used in a dilution series. Two culture media with different C and N sources were employed: Malt Extract Agar (MEA) and Czapek's Agar (CzA) (Sigma-Aldrich Inc., St. Louis, USA). Streptomycine (Spectrum Chemical Mfg. Corp., Gardens, USA) was added to each medium (100 µg/ml) to suppress bacterial growth. Soil suspension in an amount of 0.5–1 mL from the dilutions 1:10–1:100 (soil:sterile water) was mixed with the agar medium at 40 °C in Petri dishes (90 mm diameter). The plates were incubated at 25 °C (all samplings), 37 °C (March 2011 and August 2011), and 45 °C (August 2011) in darkness for 7–14 days (2–3 plates for each medium and temperature).

After incubation, the emerging fungal colonies were transferred to MEA and CzA for purification and further taxonomic identification. In an attempt to induce sporulation, all non-sporulating isolates were also grown on Oatmeal Agar (Sigma-Aldrich Inc., St. Louis, USA) as recommended by Bills et al. [32], and on Water Agar (agar: 20 g, water: 1000 mL). Taxonomic identification was based on morphological characteristics of fungal isolates. All names of the identified species are cited according to the database of Kirk et al. [33].

### 2.5. Data analysis

Density of fungal isolates was expressed as “colony forming units” (CFU) per g dry substrate. Analysis of diversity was based on the Shannon-Wiener index (*H*) and evenness (*J* = *H*/*H*_max_) [34].

To analyze spatial and seasonal variations in the microfungal community structure, two major groupings were selected: mesic *Penicillium* spp. and xeric melanin-containing microfungi; in the latter grouping, species with large multi-cellular spores and species forming picnidial fruit bodies were examined separately because of their abundance. The contribution of each group to the mycobiota structure was estimated as an average of its density (number of isolates of a particular group in the sample/total number of all isolates in the sample, i.e., its relative abundance in total isolate number in the sample) and its relative abundance (percentage) in the Shannon index. We used the latter characteristic together with direct density because (i) it is logarithmic, thus preventing an overestimation of heavily sporulating species, and (ii) it takes into account not only the number of isolates but also the number of species comprising the aforementioned groupings.

Statistical analysis was conducted using XLSTAT (http://www.xlstat.com). A one-way ANOVA followed by multiple-comparison tests was used to compare data from different localities on edaphic parameters. We employed the non-parametric Wilcoxon signed-rank test to compare paired data from different localities and seasons on diversity characteristics, contribution of microfungal groupings, and isolate densities. To evaluate similarity between communities from different localities, the clustering of the communities based on species' relative abundance was made by the unweighted pair-group average method with Chi squared distance as the distance coefficient. A two-way unbalanced ANOVA with interactions was used to test the effect of different environmental aspects (locality position along the latitudinal and elevational gradient, locality type—open or under shrubs, and season), separately and in interaction, on the above mycological parameters. The relationship of diversity characteristics, contribution of microfungal groupings, and isolate densities with organic matter content, moisture content, electrical conductivity, and pH was estimated by linear regression analysis.

## 3. Results

### 3.1. Edaphic characteristics

Expectedly, in the summer, moisture content in the studied soils was lower (1.3–2.3-fold), with less expressed spatial variations (Table 1). The Hatzeva and Sapir localities were the driest and the wettest, respectively, in both seasons. Summer temperatures reached more than 53 °C in the soil of sun-exposed localities. The Arava soils are slightly alkaline, with minimal and maximal pH in the Elifaz sun-exposed and the Sapir shady localities, respectively (Table 1). Organic matter content was expectedly significantly higher under shrubs (*Nitraria retusa*) in comparison with the sun-exposed localities, with minimal and maximal values recorded in the Elifaz sunny and Sapir shady soils, respectively (Table 1). Similarly, electrical conductivity was remarkably higher in the shrub localities; maximal values for both locality types were registered in the Sapir soils (Table 1). According to the established relationship between EC values and salinity classes [35], the majority of the Arava soil samples can be considered non-saline (EC < 2.0 dS/m), while the soil of the Sapir shrub localities is moderately saline (4.1–8.0 dS/m).

**Table 1.** Selected edaphic parameters (mean ± SD) in different localities of the Arava Valley, Israel. Means with the same letters are not significantly different (a one-way ANOVA followed by multiple comparisons test, at the 5% level).

Locality	Moisture content, % (n = 6)		Temperature, °C (n = 6)^1^		pH (n = 3)^2^	Organic matter, % (n = 3)^b^	Electrical conductivity, dS/m (n = 3)^b^
	Summer 2014	Winter 2015	Summer 2014	Winter 2015			
Elifaz, open	0.3 ± 0.1b	0.7 ± 0.3bc	48.9 ± 2.9	23.8 ± 2.4	7.4 ± 0.1b	0.16 ± 0.02c	0.52 ± 0.26b
Elifaz, shrubs	0.4 ± 0.1b	0.8 ± 0.2bc	42.0 ± 2.7	16.8 ± 1.4	7.5 ± 0.06ab	0.73 ± 0.1b	1.29 ± 0.16ab
Sapir, open	0.6 ± 0.2ab	1.2 ± 0.2ab	52.5 ± 1.4	25.5 ± 1.0	7.6 ± 0.15ab	0.37 ± 0.18b	1.25 ± 1.0b
Sapir, shrubs	0.7 ± 0.3ab	1.6 ± 0.6a	43.2 ± 2.1	22.1 ± 0.7	7.8 ± 0.2a	1.91 ± 0.96a	6.0 ± 0.74a
Hatzeva, open	0.3 ± 0.05b	0.5 ± 0.1c	53.4 ± 2.3	22.8 ± 1.0	7.6 ± 0.1ab	0.37 ± 0.14b	0.31 ± 0.06c
Hatzeva, shrubs	0.3 ± 0.1b	0.4 ± 0.05c	42.3 ± 2.2	20.5 ± 0.6	7.7 ± 0.2a	0.74 ± 0.21ab	1.45 ± 1.3b

^1^ measured during the hours of 9–10 a.m. (Elifaz), 12 a.m.–1 p.m. (Sapir), and 2–3 p.m. (Hatzeva); ^2^ winter samples.

### 3.2. Composition and diversity of microfungal communities

In the course of the study (March 2011–February 2015), 198 species were isolated and identified. They included Zygomycota (10 species), teleomorphic Ascomycota (38), anamorphic Ascomycota (149), and Basidiomycota (1). Eleven strain types remained non-sporulating in culture and were not identified. The identified species belonged to 84 genera. The most numerous were *Aspergillus* and *Penicillium* (23 species each), *Chaetomium* (18), *Alternaria* and *Phoma* (7 each), and *Curvularia* (5).

From summer 2014 and winter 2015 samplings, 64 and 84 species were isolated, respectively; 39 species were isolated in both seasons. Main seasonal differences in species composition were associated with a number of species from the genera *Penicillium* (4 in summer vs. 11 in winter), *Aspergillus* (6 vs. 10), *Cladosporium* (1 vs. 4), and from the phylum Zygomycota (1 vs. 5). At the same time, the summer mycobiota contained more diverse composition of melanin-containing fungi with large multi-cellular spores—19 species in comparison with 13 species in the winter mycobiota.

The open microfungal communities at each sampling site, both in summer and winter, were characterized by significantly higher species richness than the communities from shrub localities (Wilcoxon signed-rank test, *p* < 0.05), with differences more expressed in the summer (*p* < 0.001, except for Hatzeva with *p* < 0.05) (Table 2). Open communities from the soil near Elifaz had a significantly higher number of species in comparison with the corresponding communities of those located northward, from Sapir and Hatzeva (Wilcoxon test, *p* < 0.05); for the shrub communities, significant differences were found only between the most distant Elifaz and Hatzeva communities (Wilcoxon test, *p* < 0.05). Seasonal differences in species richness were significant only between the Elifaz open communities as well as between the Sapir shrub communities (richer in the winter in both cases; Wilcoxon test, *p* < 0.05). Spatial and seasonal variations in heterogeneity (Shannon index) and especially in evenness of the microfungal communities were less obvious than in species richness (Table 2). The most pronounced differences in diversity characteristics were found in the soil near Sapir in the center of the Arava Valley, with open communities being more heterogeneous and even than the shrub communities (Wilcoxon test, *p* < 0.05), especially in winter (*p* < 0.01).

**Table 2.** Diversity characteristics of soil microfungal communities (average ± SD, n = 6; *S*: number of species, including sterile strains, *H*: Shannon index, *J*: evenness) in different localities of the Arava Valley, Israel.

Locality	Summer 2014				Winter 2015		
	*S*	*H*	*J*		*S*	*H*	*J*
Elifaz, open	18 ± 2 (40^1^)	1.88 ± 0.08	0.66 ± 0.01		20 ± 2 (44)	1.76 ± 0.43	0.59 ± 0.14
Elifaz, shrubs	12 ± 2 (27)	1.67 ± 0.16	0.68 ± 0.05		14 ± 3 (35)	1.84 ± 0.37	0.70 ± 0.12
Sapir, open	17 ± 3 (38)	1.81 ± 0.13	0.65 ± 0.05		16 ± 3 (42)	1.88 ± 0.34	0.68 ± 0.1
Sapir, shrubs	11 ± 2 (24)	1.35 ± 0.15	0.57 ± 0.07		13 ± 1 (29)	1.41 ± 0.37	0.54 ± 0.11
Hatzeva, open	15 ± 3 (34)	1.67 ± 0.29	0.63 ± 0.07		14 ± 3 (34)	1.91 ± 0.36	0.74 ± 0.11
Hatzeva, shrubs	11 ± 3 (21)	1.51 ± 0.24	0.64 ± 0.07		11 ± 2 (20)	1.54 ± 0.35	0.65 ± 0.11

^1^ in parenthesis: overall number of species.

**Figure 2.**
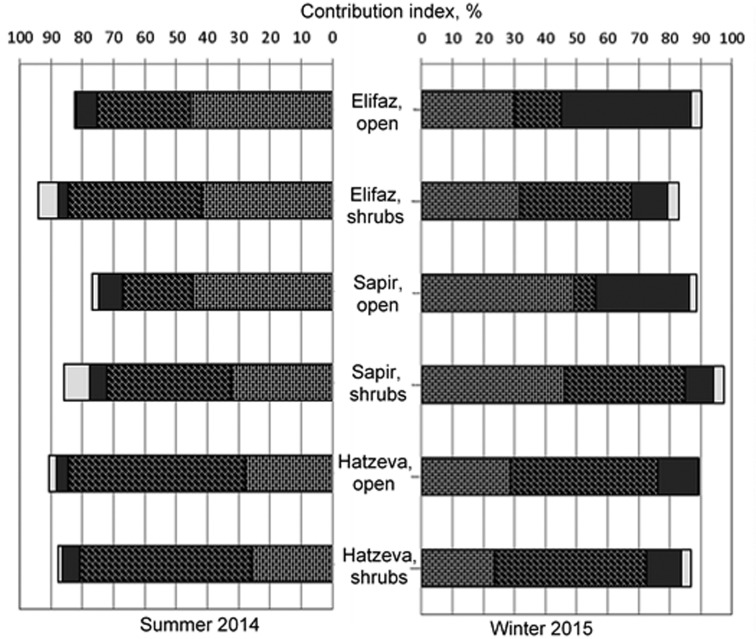
Contribution of melanin-containing species (
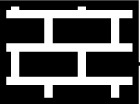
—with large multi-cellular spores; 
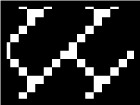
—with picnidial fruit bodies; 
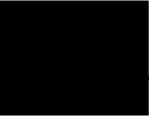
—others) and *Penicillium* spp. (
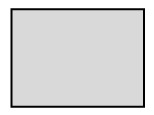
) to communities' structure in the soil of the Arava Valley, Israel.

The main core of microfungal communities in all localities studied during the whole period of the investigation was composed by melanin-containing species (Table 3, Figure 2); they comprised 51.5% (102 species) of general mycobiota composition and 51.7–99% of the contribution index in the soil of different localities. Among the most frequently and abundantly occurring microfungi, species with large, thick-walled, and many-celled conidia—such as *Alternaria atra*, *A. alternata*, *A. phragmospora*, and *Monodyctis fluctuata*, were found (14.5–70.6% of the contribution index) as well as picnidial fungi—*Boeremia exigua*, *Coleophoma empetri*, and *Phoma medicaginis* (7.2–73.7%); in the winter communities, especially from the open soil, a high abundance of *Cladosporium cladosporioides* was also recorded (Table 3). No consistent pattern was revealed in spatial variation of either melanin-containing species on a whole or melanics with large multi-cellular spores; differences between open and shrub localities were mostly non-significant. In the summer, communities from the soil near Hatzeva were characterized by the lowest contribution of melanized fungi with large multi-celled spores (Wilcoxon test, *p* < 0.01). At the same time, the Hatzeva communities contained the highest abundance of picnidial species (Wilcoxon test, *p* < 0.05 or *p* < 0.01), especially in open localities, with differences from other sites mostly pronounced in summer (Wilcoxon test, *p* < 0.01).

With respect to seasonal changes, the summer and winter open and, to a lesser extent, shrub communities significantly differed in contribution of the aforementioned groups of melanin-containing species (Wilcoxon test, *p* < 0.05 or *p* < 0.01), except for the Hatzeva communities, which differed significantly only in abundance of picnidial species (Wilcoxon test, *p* < 0.05) (Figure 2). Most values for melanin-containing fungi were higher in the summer, while in the soil near Sapir, the seasonal trend for all melanized species and melanics with large multi-cellular spores was opposite.

**Table 3.** Most frequent microfungi from different localities of the Arava Valley, Israel, with their relative abundance (%). Melanin-containing species are underlined.

Species	Summer 2014						Winter 2015					
	Elifaz		Sapir		Hatzeva		Elifaz		Sapir		Hatzeva	
	open	shrubs	open	shrubs	open	shrubs	open	shrubs	open	shrubs	open	shrubs
Zygomycota												
*Rhizopus arrhizus*	0.45	0.9	0.3	0.07	0.3	−	0.8	1.2	1.3	0.03	0.2	1.1
teleomorphic Ascomycota												
*Ch. succineum*	0.08	−	−	0.3	−	−	0.05	−	−	−	0.1	−
*Chaetomium* sp.	0.2	0.09	0.4	0.02	0.08	−	0.15	−	−	0.4	−	−
*Chaetomium* sp.1	−	−	−	−	−	−	−	0.06	0.4	1.4	−	−
*Sporormiella minima*	0.55	−	1	−	−	−	−	0.25	0.6	−	−	−
anamorphic Ascomycota												
*Alternaria alternata*	8.3	8.2	4	1.3	0.9	1	7.3	9.6	18.7	1.3	4.9	3.2
*A. atra*	41.5	23.1	38.1	25.4	24.6	18.9	19.9	13.8	33.6	39.6	19	17.2
*A. chlamydospora*	-	-	-	0.02	-	-	-	-	0.5	3.9	1	0.35
*A. phragmospora*	0.6	0.15	6	0.25	0.1	0.3	0.05	1.3	1.9	0.7	1.3	
*Aspergillus fumigatus*	0.7	0.2	10.4	8.2	0.6	.250	0.5	10.8	0.2	0.03	-	-
*A. niger*	0.85	.4	0.2	-	0.15	0.08	1.9	1.1	1.8	0.4	0.7	-
*A. ustus*	−	0.05	−	−	−	0.15	0.4	1.1	−	0.1	0.3	−
*Botryotrichum piluliferum*	0.2	0.05	−	−	0.8	−	−	−	0.2	−	−	−
*Boeremia exigua*	13.6	29.8	10.7	8.8	44.4	24	8.5	28.4	0.8	4.1	24	10.5
*Cladosporium cladosporioides*	0.4	0.09	1	0.05	1	0.3	44.2	7.4	24	1.6	8.1	4
*Cochliobolus spicifer*	−	−	−-	−	−	−	0.1	−	0.4	0.07	0.1	−
*Coleophoma empetri*	9.8	18	8.4	37	10.8	34.4	3.8	13.8	3	40	25	49.4
*Exserohilum rostratum*	−	−	−-	−	0.04	−	−	−	0.1	−	0.1	0.25
*Epicoccum nigrum*	−	0.2	0.3	−	0.04	−	−	−	0.2	0.03	−	−
*Fusarium equiseti*	10.9	.09	10.1	5.6	4	3.5	2.9	2	0.5	0.9	5.5	7.8
*F. oxysporum*	1.6	1.5	−	−	0.1	−	0.15	0.3	1.7	0.1	.2	0.1
*F. sporotrichioides*	0.55	−	0.8	−	0.3	0.03	−	−	0.4	−	−	−
*Monodictys fluctuata*	1	9.4	0.6	0.2	0.6	3.7	0.8	5	0.1	0.7	.5	−
*Myrothecium roridum*	0.08	−	−	0.8	0.08	4.5	0.05	0.1	−	−	0.7	0.55
*M. verrucaria*	0.15	0.6	−	−	0.08	−	1.1	0.6	−	−	−	−
*Neocucurbitaria cava*	0.08	−	0.15	0.02	0.1	0.08	−	−	−	−	0.4	−
*Papulaspora pannosa*	1.3	0.09	0.15	−	0.8	−	−	0.06	0.1	0.07	0.8	−
*Penicillium aurantiogriseum*	0.2	5	1	4.2	1.1	0.8	−	−	0.7	1.6	−	2
*Phoma medicaginis*	4.4	0.2	1.8	0.05	8.6	4.8	−	0.4	0.5	0.3	2.9	1
*Pleospora tarda*	−	0.7	0.7	−	0.3	−	0.9	0.2	1.2	1.9	−	−
*Stachybotrys chartarum*	0.4	0.4	1.1	−	0.08	3	0.6	0.05	1.2	0.03	0.7	0.2
*Westerdykella**capitulum*	−	−	0.07	−	0.08	−	−	−	0.4	−	0.1	−

*Penicillium* spp. constituted the minor component of all microfungal communities; they comprised 0–14.4% of the contribution index. No clear trend in spatial and seasonal distribution was found for penicillia. Only in summer, in the shrub communities near Sapir and Elifaz, an abundance of *Penicillium* spp. was predictably higher than in the open communities (Wilcoxon test, *p* < 0.05) (Figure 2).

**Figure 3.**
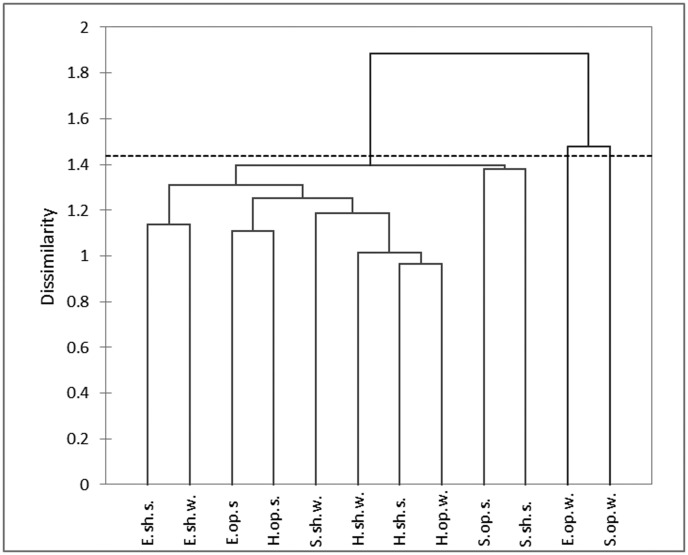
Clustering the microfungal communities from different localities (E: Elifaz, S: Sapir, H: Hatzeva; op.: open, sh.: under shrubs) and seasons (w.: winter, s.: summer) of the Arava Valley, Israel, based on species relative abundance.

Clustering the microfungal communities based on species relative abundances showed that the summer communities were more similar to each other than the winter communities (Figure 3). Communities from the soil of shady localities comprised a more homogeneous group than those from sunny localities. Among different sites, the communities from the Hatzeva soil were more closely related to each other. In most cases, the communities from the same locality type (open or under shrubs) were more similar to each other than the communities from open and shrub localities at the same sampling area.

### 3.3. Thermotolerant mycobiota

At 37 °C, 44 species were isolated, with 17 species belonging to teleomorphic Ascomycota. *Aspergillus* (10 species) and *Chaetomium* (9 species) were the most numerous genera. Nearly half (19) of the species were isolated only at 37 °C. The composition of microfungal communities at 37 °C was almost entirely different from that at 25 °C. Melanin-containing species dominant at 25 °C did not grow at 37 °C, and *Aspergillus fumigatus*, *A. niger*, *Canariomyces notabilis*, *Chaetomium strumarium*, *Ch. nigricolor*, *A. nidulans*, and *Rhizopus arrhizus* prevailed or frequently occurred. At 45 °C, poor species composition was revealed: *C. notabilis*, *Ch. strumarium*, *A. fumigatus*, *Canariomyces thermophile*, *Ch. nigricolor*, *A. nidulans*, *A. crustosus*, *Papulaspora pannosa*, *Thermomyces lanoginosus*, *Humicola thermophila*, *Neocucurbitaria cava*, and *Westerdykella capitulum*, a total of 12 species, with the first three species being most frequently isolated.

### 3.4. Density of microfungal isolates

**Figure 4.**
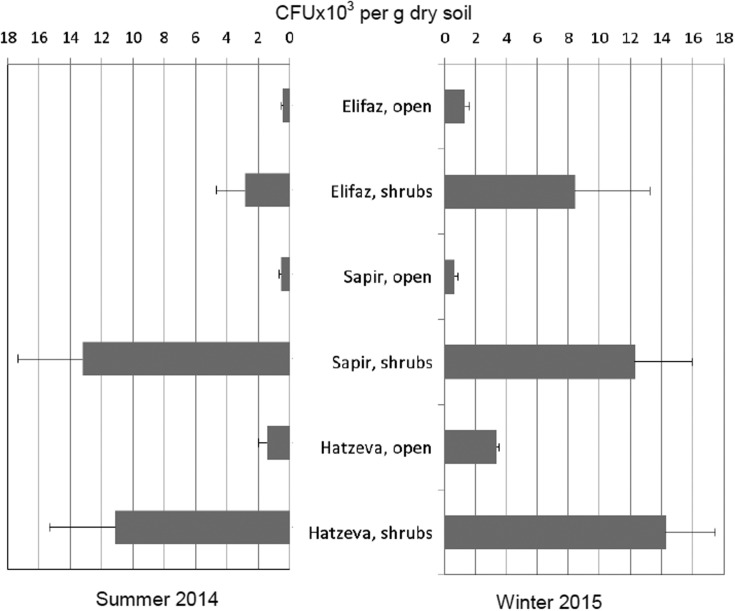
Density of microfungal isolates in the soil of different localities at the Arava Valley, Israel. Horizontal bars represent standard deviations (n = 6).

Density of microfungal isolates was mostly sensitive to spatial and seasonal variations. Both in summer and winter, the number of CFU was highly significantly lower in the soil of open localities than under shrubs (Wilcoxon test, *p* < 0.01) (Figure 4). In both seasons, among open communities, the highest isolate density was recorded in the northern part of the Arava, near Hatzeva (Wilcoxon test, *p* < 0.01); differences among the shrub communities were less pronounced. The summer open communities held significantly lower CFU numbers than the winter open communities (Wilcoxon test, *p* < 0.05 or *p* < 0.01); again, seasonal variations in the communities from the shrub localities were less expressed.

### 3.5. Comparison between microfungal communities from the soil near Hatzeva (the Arava Valley) and near Makhtesh Ramon (Central Negev)

Microfungal communities from the soil near Hatzeva, both sun-exposed and shrubby, and both in summer and winter, were significantly poorer on species composition than the communities near Makhtesh Ramon, the area located at a close latitudinal position (near 15 km southward from Hatzeva) but at a height of 200–220 m a.s.l (Wilcoxon test, *p* < 0.05 or *p* < 0.01). In the Hatzeva communities, melanin-containing species on a whole and, especially picnidial species, were significantly more abundant (Wilcoxon test, *p* < 0.05 or *p* < 0.01) while the contribution of melanics with large multi-cellular spores was significantly higher in the soil near Makhtesh Ramon and only in summer (Wilcoxon test, *p* < 0.05 for open and *p* < 0.01 for shrub communities). In the Makhtesh Ramon shrub localities, *Penicillium* spp. were significantly more abundant (Wilcoxon test, *p* < 0.05 and *p* < 0.01 for the winter and summer communities, respectively). Notably, the density of microfungal isolates was significantly higher in the Hatzeva sun-exposed localities (Wilcoxon test, *p* = 0.05 and *p* < 0.01 for the winter and summer communities, respectively) but with significantly lower values in the shrub localities (Wilcoxon test, *p* < 0.05 and *p* < 0.01 for the summer and winter communities, respectively) as compared to the corresponding localities near Makhtesh Ramon.

### 3.6. Effect of locality type, locality position, and season on characteristics of microfungal communities

Among diversity characteristics, evenness was the least affected by the above environmental aspects, whereas species richness and, to a lesser extent, heterogeneity of microfungal communities were significantly influenced by locality type (open, sun-exposed or under canopy), locality position along the latitudinal and elevational gradient, and the interaction between them (Table 4). Among the microfungal groupings, melanin-containing species with large multi-cellular spores and picnidial fungi were significantly dependent in their distribution on each aspect separately (especially on locality type and position) and on their cumulative effect. The distribution of *Penicillium* spp. was less sensitive to the environmental aspects. Isolate density was significantly influenced both by each aspect separately (by locality type to the most extent) and the combination of locality type and position. Among the environmental aspects, both locality position and type affected the characteristics of microfungal communities in the strongest way; their interaction was also important for the distribution and structure of the communities (Table 4).

### 3.7. Effect of edaphic variables on microfungal communities

**Figure 5.**
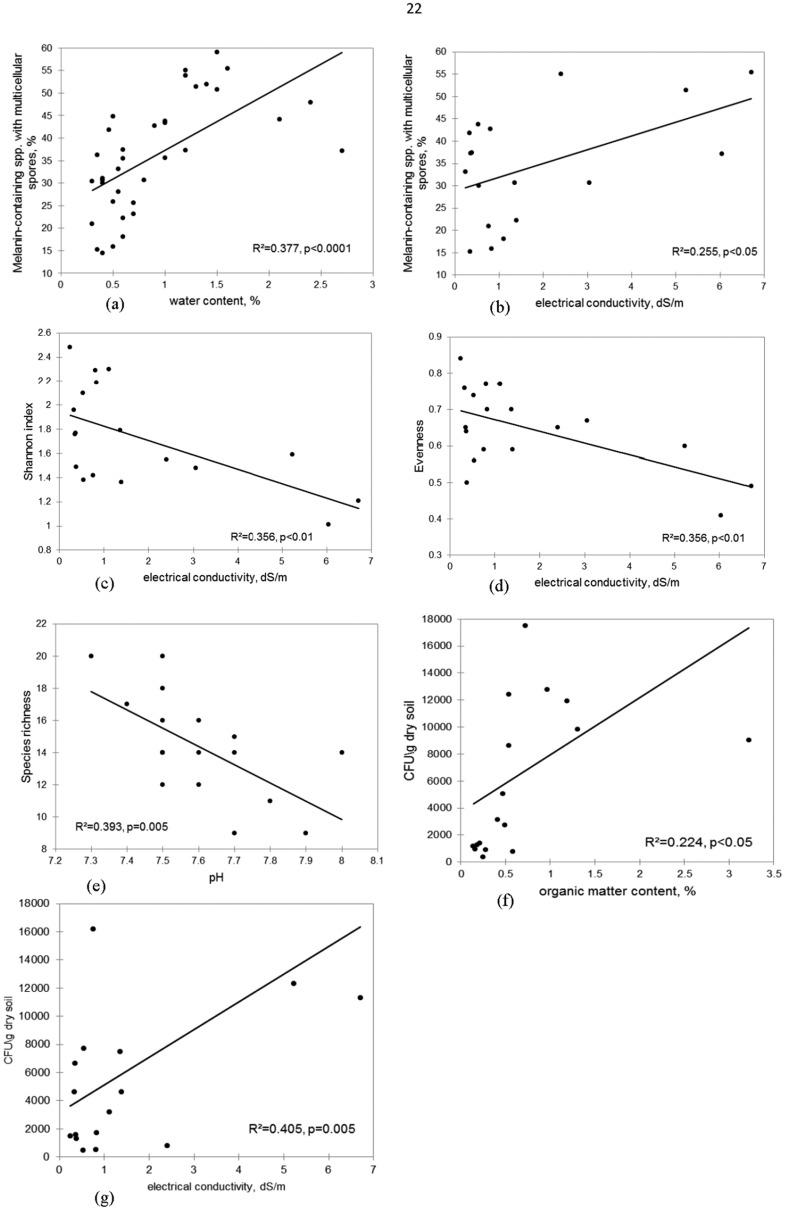
Relationship of different characteristics of microfungal communities with measured edaphic parameters in soil of the Arava Valley, Israel.

The abundance of melanin-containing species with multi-cellular spores significantly and positively correlated with moisture content in the winter (*p* < 0.0001, Figure 5a) and less strongly—with electrical conductivity (EC) (*p* < 0.05, Figure 5b). Diversity characteristics exhibited significant and negative linear relationships with EC (Shannon index and evenness—for both *p* < 0.01, Figure 5c, d) and pH (species richness: *p* = 0.005, Figure 5e). The density of microfungal isolates significantly and positively correlated with organic matter content (*p* < 0.05, Figure 5f) and more strongly—with EC (*p* = 0.005, Figure 5g). Among the measured edaphic parameters, electrical conductivity influenced the characteristics of microfungal communities in the strongest way.

**Table 4.** Data of two-way unbalanced ANOVA analysis for the effect of locality type, locality position, season, and interactions between them on different parameters of soil microfungal communities at the Arava Valley and the central Negev Desert, Israel.

Parameter	Locality type	Locality position	Season	Locality type × locality position	Locality type × season	Locality position × season	Locality type × locality position × season
Species richness	17.30****	21.09****	NS	6.33***	NS	NS	NS
Shannon index	11.28***	3.97^@^	NS	2.76^@^	NS	NS	NS
Evenness	NS	NS	NS	3.96^@^	NS	NS	NS
Melanin-containing spp.	NS	5.59****	NS	NS	NS	4.81**	NS
Melanized spp. with multicellular spores	11.29***	8.86****	7.03*	4.26*	NS	8.51****	NS
Picnidial spp.	43.71****	41.34****	9.8**	2.88^@^	NS	NS	NS
*Penicillium* spp.	17.29****	6.97***	NS	4.79*	NS	3.07^@^	NS
Isolate density	12.19***	3.59^@^	4.12^@^	4.08*	NS	NS	NS

^@^ ≤ 0.05; * ≤ 0.01; ** ≤ 0.005; *** ≤ 0.001; ****≤ 0.0001.

## 4. Discussion

The soils of the Arava Valley are inhabited by comparatively rich diversity of culturable microfungi—198 identified species. The data showed once again that, in spite of climatic hostility, desert soils which characterized by high spatiotemporal heterogeneity in resource availability can maintain diverse microfungal biota [16],[17]. An analogous pattern was found along the precipitation gradient in Israel for another group of soil microorganisms, bacteria, which the overall taxonomic diversity at the species level in the arid site was high and similar to those found in the humid Mediterranean, Mediterranean, and semi-arid sites [36].

### 4.1. Variations in density of microfungal isolates

The density of microfungal isolates was subjected to pronounced spatial and seasonal changes in soils of the Arava Valley. This quantitative parameter, which can be considered an indirect characteristic of fungal biomass, expectedly displayed a significant positive linear relationship with organic matter (OM) content. Isolate density also strongly, positively correlated with soil electrical conductivity (EC). Likewise, EC is known to positively correlate with OM content [37]. On a whole, EC in a definite interval can serve as an indirect indicator of various edaphic conditions because it is also closely associated with the amount of dissolved salts, water holding capacity, soil texture, and porosity [38]. Perhaps these associations can explain the fact that in comparison with other measured edaphic parameters, EC affected the characteristics of the Arava microfungal communities most efficiently (Figure 5).

### 4.2. Variations in diversity characteristics

Unlike isolate density, heterogeneity and evenness of the microfungal communities exhibited a significantly negative relationship with EC. Species richness, which significantly and negatively correlated with pH, also displayed the opposite to isolate density spatial distribution, which was significantly higher under shrubs than in open localities. A similar pattern has been revealed at the western Negev desert where in the most organic-rich and moss-dominated crusts harboring the highest density of microfungal isolates, the number of species and diversity level were lower than in the cyanobacterial crusts [19]. The negative relationship between the diversity level and the CFU number was expected. Often, an increase in the isolate density is caused by an abundant development of one or two species, which decrease both species richness and the diversity level of the microfungal communities.

### 4.3. Variations in structure of microfungal communities

Expectedly, melanin-containing species comprised more than half of species composition and dominated all microfungal communities in the Arava soils (Figure 2). These species are well known stress-tolerant microorganisms resistant to solar and UV radiation, high temperature, desiccation, oligotrophic conditions, and chemical and radioactive pollution ([39] and references therein). A dominance of dark-colored microfungi is typical for almost all mycologically studied desert soils [5],[7],[9],[11],[12],[15],[40],[41]. The prevalence of melanized fungi with large multi-cellular spores in nearly all areas of the Negev Desert [4],[18]–[21] was also characteristic for most studied localities of the Arava Valley. Such species (from the genera *Ulocladium* and *Alternaria*) were the most widespread in the soils of the Atacama Desert, which is known as one of the driest locations on Earth [8]. Likewise, these fungi, belonging to the order Pleosporales, were found to predominate in the biological soil crusts studied by the culture-independent molecular approaches at the Colorado Plateau, in the semi-arid grassland in central New Mexico, and in the southwestern deserts of the USA [6],[42],[43] as well as in the crusted sand of the Tennger Desert, China [14]. The aforementioned studies along with our survey at the Arava Valley confirm once again that dark pigmentation together with multi-cellular spore morphology are very important features for dispersal and resting functions of soil microfungi in climatically stressful desert habitats.

Another group of melanin-containing species, which frequently and abundantly occurred in the Arava soils, produces comparatively small (5–15 µm long) light-colored conidia inside the dense multi-layered picnidial fruit bodies. Similar to the melanized fungi with large multi-celled spores, these picnidial fungi are known also as phylloplane-inhabiting species [44],[45], and thick-walled dark-brown or black spherical fruit bodies provide them with protection against extremely stressful and highly fluctuating environmental conditions.

Notably, in the soil near Hatzeva, the contribution of melanin-containing fungi with large multi-cellular spores was highly significantly lower than in other sampling areas, while the contribution of picnidial species were significantly higher. Hatzeva is located in the northern part of the Arava Valley at an elevation of 190 m below sea level, which consequently decreases the level of UV-radiation on the soil's surface. A similar prevalence of dark-colored microfungi with small one-celled conidia (*Aspergillus niger* and *Cladosporium cladosporioides*) was characteristic for the hypersaline Dead Sea shore [46]—an extremely stressful environment but receiving very low UV-radiation because of its location at more than 400 m below sea level.

Markedly, in the soil near Sapir, an abundance of melanized species with many-celled spores was lower in the summer than in the winter, while in other sampling areas, the opposite trend was predictably observed. Possibly, it was due to the comparatively abundant development of the thermotolerant *Aspergillus fumigatus* in the soil of the open Sapir localities in the summer (8–23% of microfungal isolates at 25 °C). Whereas microfungi with dark multi-cellular conidia were unable to germinate at 37 °C, *A. fumigatus* dominated the microfungal communities both at 37 °C and 45 °C in all localities. This fungus is known as one of the most frequent and abundant thermotolerant species in a variety of desert regions [5],[9],[10],[12],[39],[47]–[49]. *A. fumigatus* together with *A. niger* and a set of teleomorphic species with perithecial fruit bodies form the main core of thermotolerant mycobiota in the region, thus showing the ability not only to survive but also to germinate and grow during a long period of high temperatures in the desert.

Distribution of microfungi with dark multi-cellular spores in the Arava Valley was subjected to remarkable spatial and seasonal variations both in abundance and composition and displayed a statistically significant positive relationship with soil moisture content in the winter. By contrast, in the southern Negev canyon, Nahal Shaharut, an abundance of these microfungi was consistently very high with no significant dependence on moisture content [18], while in the Makhtesh Ramon area (central Negev), there was a significant and negative correlation between their abundance and soil moisture [21]. Probably, extremely low moisture content in the Arava soils, even in winter and lower than what was registered in the soils of Nahal Shaharut and the Makhtesh Ramon area, resulted in such a positive relationship between the abundance of melanized microfungi with many-celled spores and soil moisture.

A comparison between microfungal communities from the northern part of the Arava Valley located at an elevation of 190 m below sea level and from the central Negev area located at a close latitudinal position but at an elevation of 200–220 m above sea level revealed significant differences. Apparently as a result of unique elevational position weakening the harmful influence of UV-radiation, the open sun-exposed localities near Hatzeva held the highest amount of microfungi but with significantly lower species richness and were characterized by the lowest and highest relative abundance of melanin-containing microfungi with large multi-cellular spores and picnidial species, respectively. In addition, the communities from the northern Arava area were the least subjected to local (open and under shrub) and seasonal (summer and winter) variations.

Importantly, cluster analysis based on the relative abundance of species revealed that, in most cases, the communities from the same locality type (open or under shrubs) were more similar to each other than to the communities from open and shrub localities at the same sampling area. The data were supported by the ANOVA analysis showing that among the environmental aspects, locality type, together with locality position along elevational and latitudinal gradients, strongly affected not only composition but also other studied characteristics of microfungal communities. A similar pattern was revealed in our study devoted to soil microfungi along the precipitation gradient in northern and central Negev [22]. It was indicated that, in most cases, the crusted and shrub localities, separated only by a few meters or less, differed in microfungal community structure much more significantly than crusted or shrub localities at a distance of tens of kilometers. This observation again confirms the fact that microclimatic and edaphic factors play an essential role in the development of soil microfungal communities, and their structure can be a sensitive indicator of changing environmental conditions at a microscale.

## 5. Conclusions

The presented study revealed the diverse culturable mycobiota inhabiting the soils of the Arava Valley along latitudinal and elevational gradients. Similar to soil mycobiotas at the Negev Desert, the Arava was dominated by stress-selected melanin-containing microfungi, but with a high abundance of not only melanized species with large multi-cellular spores but also picnidial fungi with small one-celled and light-colored conidia. The latter group significantly increased its contribution at the northern part of the Arava Valley located at 190 m below sea level. The increase in abundance of picnidial fungi in the soil of this area is apparently caused by the weakening of abiotic stress (decrease in the level of UV radiation) and the consequential weakening in the competition with melanin-containing species with large many-celled spores dominating the microfungal communities in other areas. The effect of the unique elevational position was also evident in the substantial increase of isolate density, decrease of species richness, and mitigation of both local and seasonal variations of the community characteristics. The thermotolerant mycobiota isolated at 37 °C was entirely different in its composition, being dominated by aspergilli and teleomorphic perithecial ascomycetes. The mycobiota characteristics showed a relationship of different strength and direction with the measured edaphic parameters—organic matter content, water content, pH, and electrical conductivity. Among the environmental aspects, locality type (open sunny-exposed or under shrubs) together with locality position along elevational and latitudinal gradients, strongly affected the characteristics of microfungal communities, thus demonstrating the capacity of the communities to react sensitively on a microscale's environmental variability.
